# Association between Age-Related Macular Degeneration and the Risk of Diabetes Mellitus: A Nationwide Cohort Study

**DOI:** 10.3390/biomedicines10102435

**Published:** 2022-09-29

**Authors:** Wonyoung Jung, Je Moon Yoon, Kyungdo Han, Bongseong Kim, Sungsoon Hwang, Dong Hui Lim, Dong Wook Shin

**Affiliations:** 1 Department of Family Medicine and Supportive Care Center, Samsung Medical Center, Sungkyunkwan University School of Medicine, Seoul 06351, Korea; 2 Department of Medicine, Sungkyunkwan University School of Medicine, Seoul 06351, Korea; 3 Department of Ophthalmology, Samsung Medical Center, Sungkyunkwan University School of Medicine, Seoul 06351, Korea; 4 Department of Statistics and Actuarial Science, Soongsil University, Seoul 06978, Korea; 5 Department of Clinical Research Design & Evaluation, Samsung Advanced Institute for Health Science & Technology (SAIHST), Sungkyunkwan University, Seoul 06355, Korea

**Keywords:** age-related macular degeneration, diabetes mellitus, visual disability

## Abstract

Age-related macular degeneration (AMD) is a degenerative and progressive disease of the macula, the part of the retina that is responsible for central vision. AMD shares some risk factors with diabetes mellitus (DM), but little is known about the risk of DM in individuals with AMD. With the goal of establishing novel perspectives, this study aimed to investigate the association between AMD and the risk of DM using the Korean Nationwide Health Insurance Database. Individuals aged ≥ 50 years who underwent a national health screening program in 2009 were enrolled. Participants were categorized by the presence of AMD and visual disability (VD). The Cox hazard regression model was used to examine hazard ratios (HRs) of DM with adjustment for potential confounders. Stratified analyses by age, sex, and comorbidities (hypertension or dyslipidemia) were also performed. During a mean follow-up of 8.61 years, there were 403,367 (11.76%) DM incidences among the final 3,430,532 participants. The crude HR (95% confidence interval (CI)) was 1.16 (1.13–1.20) for AMD. After adjusting for potential confounders, AMD was associated with a 3% decreased risk of DM (aHR 0.97, 95% CI 0.95–1.00), but no significant association with the risk of DM was found in AMD with VD (aHR 1.03, 95% CI 0.93–1.14). In summary, we did not find an increased risk of DM in individuals with AMD. A 3% decreased risk of DM in patients with AMD is not clinically meaningful. Our study suggests that the association between AMD and the risk of DM is weak, considering the potential confounders. Further studies examining this association are needed to extend our knowledge.

## 1. Introduction

Age-related macular degeneration (AMD) is a degenerative and progressive disease of the macula, the part of the retina that is responsible for central vision [1]. Since the number of people with AMD worldwide is expected to increase to 288 million by 2040 [2], it is important to identify comorbid conditions and health problems among those affected.

AMD is associated with traditional risk factors for cardiovascular disease, such as aging, cigarette smoking, obesity, elevated levels of cholesterol, and hypertension [3,4,5,6,7,8,9], each of which also is a risk factor for diabetes mellitus (DM). Previous studies have focused on the risk of AMD in individuals with DM [10,11,12,13], suggesting a link through chronic inflammation and oxidative stress. However, little is known about the risk of DM in people with AMD.

Notably, AMD is the leading cause of blindness in developed countries, particularly in patients over 60 years of age [2]. Visual disability (VD) is associated with a broad range of chronic conditions, including cardiometabolic, neuropsychiatric, and musculoskeletal disorders [14,15]. A nearly twofold risk of type 2 DM among people aged 65 or older with visual impairment was reported in a US claims database study [14]. In addition, a recent prospective study highlighted the role of the neighborhood environment in DM risk in people with visual impairment [16]. Environmental barriers in accessing healthcare service, proper nutrition, and recreational resources that promote positive lifestyle behaviors may contribute to the increased risk of type 2 DM in people with visual impairment. These findings suggest an association between AMD, the leading cause of visual disability, and the risk of DM. With the goal of establishing novel perspectives, we aimed to investigate the association between AMD, which is further categorized by VD status, and the risk of DM incidence using the Korean nationwide claims database.

## 2. Materials and Methods

### 2.1. Data Source and Study Setting

The National Health Insurance (NHI) in Korea is a mandatory social health insurance program and provides universal coverage to 97% of the Korean population. Due to their low-income status, the remaining 3% are covered by Medicaid, which is funded by the general Korean tax. The National Health Insurance Service (NHIS) is a public corporation that administers to both NHI enrollees and Medicaid beneficiaries. For every individual aged 40 or above, the NHIS provides a biennial national health screening that consists of a self-questionnaire on health behavior (smoking, drinking, and past medical history), anthropometric measurements (blood pressure, body mass index), and laboratory test findings (fasting glucose, serum lipid levels) [17]. Therefore, the NHIS database is an eligibility database (age, sex, disability type and severity, socioeconomic variables, income level, and type of eligibility), a medical treatment database (based on medical bills claimed by medical service providers for medical expenses), and a health screening database (results of national health screening). The NHIS database has been widely used in epidemiologic studies in Korea [18].

### 2.2. Study Population

A total of 4,470,729 participants aged  ≥50 years underwent general health screenings in 2009. To extract a homogeneous group of individuals devoid of DM or any glucose metabolism disorders, we excluded participants with DM with the following criteria: (1) history of antidiabetic medication prescription (metformin, sulfonylurea, thiazolidinedione, sodium-glucose cotransporter-2 (SGLT-2) inhibitor, dipeptidyl peptidase-4 (DPP-4) inhibitor, alpha-glucosidase, and/or insulin) before health screening in 2009 (*n* = 576,759); (2) history of type 1 DM (*n* = 37,877) or gestational DM (*n* = 58); and (3) serum fasting glucose level of 126 mg/dL or above (*n* = 188,875) at health screening in 2009. We also excluded patients who were diagnosed with DM (*n* = 17,392) or died (*n* = 13,628) within 1 year from health screening in 2009. Participants with missing data on at least one variable used in the analysis (*n* = 205,608) were also excluded. As a result, a total of 3,430,532 participants were included in the final analyses.

### 2.3. Definition of Age-Related Macular Degeneration

Individuals with AMD were identified based on the International Classification of Diseases, 10th Revision (ICD-10), code for AMD (H353) by an ophthalmologist within 1 year before the health screening examination. This operational definition of AMD has been used in previous epidemiologic studies on AMD [19,20].

### 2.4. Definition of Visual Disability

According to the National Disability Registration System (NDRS) in Korea, VD refers to a visual loss or visual field defect. Registration for disability requires submission of validated documentation of the results of disability diagnosis by a specialist physician [21]. For instance, a best-corrected visual acuity (BCVA) of 0.02 or less using the Snellen visual acuity chart is a minimum requirement to register for VD. In Korea, the severity of disability is graded from 1 (most severe) to 6 (least severe) according to predefined criteria (Supplementary Table S1), and welfare benefits, including disability pension, are determined based on that severity score [22]. Therefore, almost all individuals with disability apply for registration and are included in the National Disability Registration System in Korea.

### 2.5. Study Outcomes and Follow-Up

The study endpoint was incidence of DM, followed by the period of time analyzed. Newly diagnosed DM was defined as a prescription of antidiabetic medication that was linked to a DM ICD-10 code (E11–E14). The participants were followed from the date of health screening examination in 2009 to the date of DM incidence or death or until the end of the study period (31 December 2019), whichever came first.

### 2.6. Covariates

During the health screening, the participants provided information on lifestyle behaviors using standardized questionnaires [17]. Smoking status was categorized as non-, ex-, and current smoker. Alcohol consumption was categorized as none, mild, and heavy. Heavy drinking was defined as ≥30 g of alcohol consumption per day. Individuals were considered to have regular physical activity if they exercised strenuously ≥ 1 time/week for at least 20 min per session. Household income was dichotomized by the lowest 20 percentile according to the health insurance premium determined by income status and not by health status in the social health insurance system in Korea. Body mass index (BMI) was calculated as weight in kilograms divided by height in meters squared.

Fasting serum glucose level, serum hemoglobin level, and estimated glomerular filtration rate (eGFR) were also assessed. Hypertension was defined as any of the following: systolic blood pressure ≥ 140  mmHg, diastolic blood pressure ≥ 90 mmHg, or treatment with an antihypertensive medication that was linked to hypertension ICD-10 codes (I10–I13 and I15), and resulted in at least one claim in 1 year. Dyslipidemia was defined as total cholesterol ≥ 240 mg/dL or a history of a lipid-lowering medication that was associated with an ICD-10 code (E78). Finally, the Charlson Comorbidity Index (CCI) was calculated based on the diagnosis code [23].

### 2.7. Statistical Analysis

The comparison of baseline characteristics by the presence of AMD and VD was conducted using Student’s *t*-test for continuous variables or the chi-square test for categorical variables. The incidence rates of DM were calculated as incident cases divided by 100,000 person-years. The Cox hazard regression model was used to examine the hazard ratios (HRs) of DM. Multivariable analyses were serially adjusted for age and sex (Model 2); household income, area of residence, BMI, smoking, alcohol consumption, and regular exercise (Model 3); and fasting serum glucose level, serum hemoglobin level, eGFR, hypertension, dyslipidemia, and CCI (Model 4). These variables are based on the method used in previous studies [10,24,25,26]. P for trend was calculated among the HRs of control, AMD without VD, and AMD with VD groups. Finally, to evaluate the potential effects of modification by age, sex, and comorbidity status, P for interaction was calculated using stratified analysis.

Statistical analyses were performed using SAS version 9.4 (SAS Institute Inc., Cary, NC, USA). A *p*-value < 0.05 was considered statistically significant.

### 2.8. Ethics Statement

This study was approved by the Institutional Review Board of the Samsung Medical Center (SMC 2022-03-060). Anonymized and de-identified information was used for analyses; therefore, informed consent was not required. The database is open to all researchers whose study protocols are approved by the official review committee.

## 3. Results

### 3.1. Baseline Characteristics

A total of 3,430,532 participants were enrolled in the final analysis. At baseline, 38,726 (1.13%) participants had AMD, and 2723 (7.03% of AMD) had visual disability (Table 1). The AMD group was older with larger proportions of women and nonsmokers and had higher prevalence of hypertension and dyslipidemia compared with the non-AMD group (all *p* < 0.001). In addition, the AMD group had lower alcohol consumption, total cholesterol level, and lower income and resided less frequently in urban areas than the non-AMD group (all *p* < 0.001).

The AMD with VD group was older with larger proportions of men and current smokers and lower prevalence of hypertension and dyslipidemia compared with the AMD without VD group (all *p* < 0.001).

### 3.2. Risk of DM by AMD

During the mean 8.61 years of follow-up, there were 403,367 new cases of DM (11.76%). After considering the covariates, AMD was associated with a 3% decreased risk of DM incidence (aHR 0.95, 95% CI (0.95–1.00), p = 0.041; Table 2). Further analysis that specified AMD by VD status showed that AMD without VD was associated with a 3% lower risk for DM (aHR 0.97, 95% CI (0.94–1.00)), and no significant association with DM was found in AMD with VD (aHR 1.03, 95% CI (0.93–1.14)).

### 3.3. Stratified Analyses

**Figure 1:**
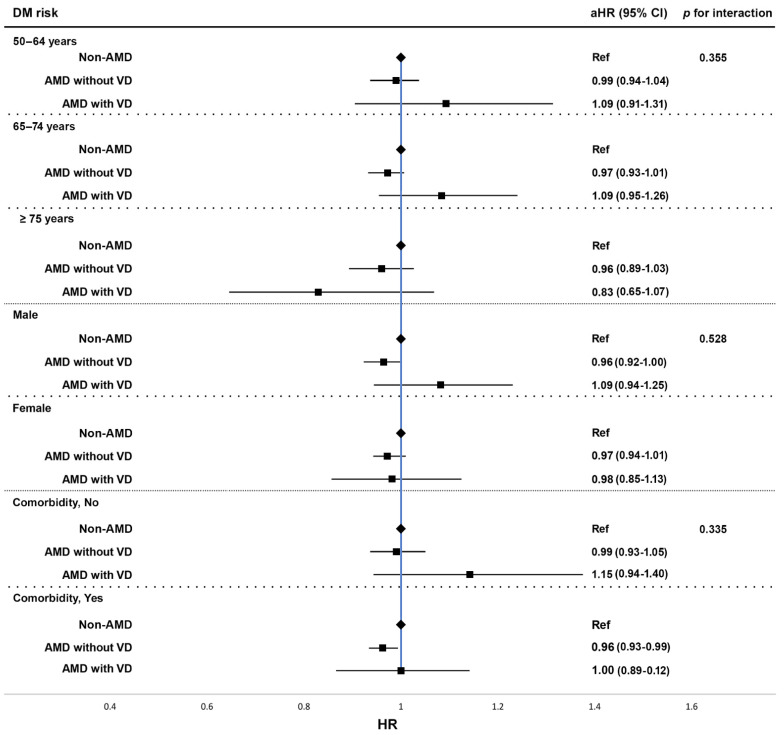
Forest plot of analyses stratified by age, sex, and comorbidity. HRs were adjusted for age, sex, income, area of residence, BMI, smoking, alcohol consumption, regular exercise, hypertension, dyslipidemia, CCI, fasting glucose level, serum hemoglobin level, and eGFR. Non-AMD group was reference (Ref). BMI, body mass index; CCI, Charlson Comorbidity Index; eGFR, estimated glomerular filtration rate; HR, hazard ratio; CI, confidence interval.

No significant interactions were found in stratified analyses according to age, sex, and comorbidities (hypertension or dyslipidemia) (Figure 1).

## 4. Discussion

Here, we investigated the association between AMD and the risk of DM. The inclusion of more than three million participants from the NHIS database enabled us to adjust for various confounders. Therefore, we had enough data to detect significant differences, even with stratified analyses. Our study revealed that AMD was associated with a 3% decreased risk of DM, which is not clinically important. No significant effect modifiers were found. Therefore, the association between AMD and the risk of DM is weak.

At baseline, the differences in demographic, socioeconomic, and lifestyle behavior variables between AMD and non-AMD group were noted. Old age, the strongest risk factor of AMD [1], and higher prevalence of hypertension, one of the moderate and consistent risk factors of AMD [1], were associated with AMD in univariate analysis. Large proportions of nonsmokers and nondrinkers and less frequent urban residency in the AMD group may be attributed to older age in the AMD group (67.06 ± 8.56 years) than the non-AMD group (60.09 ± 8.19 years). For total cholesterol level, although the difference was significant (*p* < 0.001) due to the large sample size, it was not clinically important. The smaller proportion of those in the lower 20% income in the AMD without VD group than the AMD with VD group can be explained by the limited access to healthcare service.

There are several pathophysiological mechanisms that suggest a possible link between AMD and DM. Chronic inflammation and oxidative stress may explain the link between AMD and the risk of DM. Oxidative stress triggers outer blood retinal barrier degeneration that leads to AMD [27,28], and oxidative stress is a causative factor in the development of insulin resistance [29,30]. In vitro overstimulation of interleukin 17 receptor C (IL17RC), of which a high level is detected on the surface of peripheral blood cells from patients with AMD, was shown to cause PI3K/Akt/GSK3 insensitivity, high GSK3 activity associated with insulin resistance, and type 2 DM [31]. A transcriptome-wide association study found that the BCAR1, CFDP1, and TMEM170A genes overlap with significant genome-wide association study signals of type 2 DM [32], supporting the potential link between AMD and DM. In addition, the two diseases share common risk factors, such as aging, obesity, and unhealthy lifestyle [33,34].

To our knowledge, this is the first study to report DM risk in people with AMD. Several epidemiologic studies have focused on the risk of AMD in DM patients, and the results of those studies are inconsistent [10,11,12,13,26,35,36]: higher risk of neovascular AMD [11] or late AMD [12] was reported in some studies, whereas no increased risk of AMD [13,35] was reported in another. Here, our study suggests a slightly lower risk of DM in AMD patients, contrary to our expectations. The reason for this negative association is not clear; however, one possible explanation is that about 17% of participants with a previous history of DM were excluded from our study population. Excluding participants with a DM diagnosis before baseline decreased the likelihood of individuals with AMD who were 50 years of age or older developing DM, as participants with a high likelihood of developing DM were eliminated before analysis. A report from using the Korean NHIS database (2003–2012) showed that the average age at diagnosis of DM was 64 ± 15 years [37]. According to the Korean Retina Society, the average age at diagnosis of AMD was 69.7 ± 8.0 years [38].

Since the association between AMD and DM was nullified in our final Model 4 in which covariates were serially adjusted from Model 1 (crude), other confounders may explain the association. Therefore, shared risk factors among AMD and DM may explain the association between AMD and DM rather than a common pathophysiology.

Another possible explanation is that lifestyle modification (smoking cessation, physical activity, diet, and nutritional support) prescribed in individuals with AMD may have preventive effects on the development of DM [39]. However, these observations do not fully explain the null or even negative association, and further studies are needed to replicate our finding and to investigate pathophysiological mechanisms.

It is also noteworthy that AMD patients with VD have a slightly higher risk of DM than AMD patients without VD (aHR 1.03, 95% CI (0.93–1.14); aHR 0.97, 95% CI (0.94–1.00), respectively). The strength of our study includes the use of the NDRS in our analysis. VD is associated with an increased risk of DM [14,15], regardless of AMD pathogenesis. Visual impairment is associated with physical inactivity [40,41,42], which may have adverse consequences for people with VD. Further functional consequences, including social isolation, daily activity restriction, poor quality of life, and frailty, can also result from visual impairment [43,44,45,46,47,48,49,50], especially in the elderly population. Limited access to information and healthcare facilities in people with visual impairment [51] may also contribute to this association. In addition, visual impairment significantly affects nutritional status, with a higher prevalence of obesity [52]. Further studies are needed to explain these findings.

Several limitations should be considered in interpreting our results. First, AMD might be underdiagnosed, especially during the early stages. Most AMD patients in Korea are diagnosed early without symptoms that can be identified from the claims database. Since we used the operational definition based on an ICD-10 code and not retinal photographs, the association between AMD and DM might be weaker in our findings than the actual association. Therefore, unmeasured confounders could be possible. Second, our results cannot be extended to other ethnic groups, considering the ethnic differences in genetic factors related to AMD [53,54]. Finally, other possible confounders, such as nutrition and dietary factors [55,56], were not controlled.

In summary, we did not find an increased risk of DM in individuals with AMD. Our study suggests that the association between AMD and the risk of DM is weak, considering the potential confounders. Further studies examining this association are needed to extend our knowledge.

## Figures and Tables

**Table 1:** Baseline characteristics of the study population according to the presence of age-related macular degeneration.

	Age-Related Macular Degeneration			Age-Related Macular Degeneration		
	Absent (*n* = 3,391,806)	Present (*n* = 38,726)	*p*-Value	Without VD (*n* = 36,003)	With VD (*n* = 2723)	*p*-Value
Mean age, years	60.09 ± 8.19	67.06 ± 8.56	<0.001	67.02 ± 8.57	67.61 ± 8.50	<0.001
Sex, male	1,588,565 (46.8)	15,889 (41.0)	<0.001	14,623 (40.6)	1266 (46.5)	<0.001
Smoking			<0.001			<0.001
Nonsmoker	2,294,086 (67.6)	28,673 (74.0)		26,724 (74.2)	1949 (71.6)	
Ex-smoker	519,477 (15.3)	5950 (15.4)		5515 (15.3)	435 (16.0)	
Current smoker	578,243 (17.1)	4103 (10.6)		3764 (10.5)	339 (12.5)	
Alcohol consumption			<0.001			<0.001
None	2,208,593 (65.1)	29,238 (75.5)		27,174 (75.5)	2064 (75.8)	
Mild	978,072 (28.8)	8041 (20.8)		7495 (20.8)	546 (20.1)	
Heavy	205,141 (6.1)	1447 (3.7)		1334 (3.7)	113 (4.2)	
Regular physical activity	709,047 (20.9)	7861 (20.3)	0.004	7295 (20.3)	566 (20.8)	0.012
Anthropometrics						
Body mass index, kg/m^2^	23.94 ± 2.95	23.85 ± 2.99	<0.001	23.85 ± 2.99	23.80 ± 3.01	<0.001
WC, cm	81.57 ± 8.19	82.05 ± 8.23	<0.001	82.02 ± 8.22	82.46 ± 8.29	<0.001
Systolic BP, mmHg	125.82 ± 15.68	127.55 ± 15.68	<0.001	127.58 ± 15.66	127.24 ± 15.88	<0.001
Diastolic BP, mmHg	77.76 ± 10.14	77.54 ± 9.94	<0.001	77.55 ± 9.93	77.48 ± 10.07	0.052
Comorbidity						
Hypertension	1,414,845 (41.7)	21,292 (55.0)	<0.001	19,851 (55.1)	1441 (52.9)	<0.001
Dyslipidemia	857,795 (25.3)	11,906 (30.7)	<0.001	11,100 (30.8)	806 (29.6)	<0.001
Laboratory findings						
Glucose, fasting, mg/dL	94.63 ± 11.55	94.58 ± 11.47	0.463	94.58 ± 11.48	94.68 ± 11.36	0.628
eGFR, mL/min/1.73 m^2^	83.13 ± 33.27	80.71 ± 35.12	<0.001	80.70 ± 35.16	80.78 ± 34.53	<0.001
Total cholesterol, mg/dL	202.30 ± 37.37	201.40 ± 37.94	<0.001	201.47 ± 37.94	200.39 ± 37.91	<0.001
Triglycerides ^a^, mg/dL	117.49 (117.43–117.56)	116.97 (116.38–117.55)	0.227	116.81 (116.21–117.42)	119.03 (116.80–121.31)	0.049
HDL-C, mg/dL	56.13 ± 31.35	55.95 ± 33.58	0.072	56.09 ± 33.92	54.13 ± 28.62	0.004
LDL-C, mg/dL	120.70 ± 38.57	120.79 ± 38.57	0.665	120.79 ± 38.37	120.75 ± 41.21	0.898
Hemoglobin, mg/dL	13.67 ± 1.45	13.41 ± 1.42	<0.001	13.40 ± 1.42	13.43 ± 1.41	<0.001
Urban residency	1,545,555 (45.6)	15,781 (40.8)	<0.001	14,664 (40.7)	1117 (41.0)	<0.001
Income of lowest 20%	727,814 (21.5)	6931 (17.9)	<0.001	6407 (17.8)	524 (19.2)	<0.001
CCI	1.00 ± 1.15	1.47 ± 1.35	<0.001	1.47 ± 1.35	1.49 ± 1.33	<0.001

Data are expressed as the mean ± standard deviation or number (%). VD, visual disability; WC, waist circumference; BP, blood pressure; eGFR, estimated glomerular filtration rate; HDL-C, high-density lipoprotein cholesterol; LDL-C, low-density lipoprotein cholesterol; CCI, Charlson Comorbidity Index. ^a^ Geometric mean (95% confidence interval).

**Table 2:** Association between age-related macular degeneration and the risk of diabetes mellitus according to visual disability status.

	Subjects (N)	Case (*n*)	IR Per 1000 Person-Years	Model 1 (Crude) HR (95% CI)	Model 2 aHR (95% CI)	Model 3 aHR (95% CI)	Model 4 aHR (95% CI)
Non-AMD	3,391,806	398,308	13.6	1 (Ref.)	1 (Ref.)	1 (Ref.)	1 (Ref.)
AMD	38,726	5059	15.8	1.16 (1.13–1.20)	1.00 (0.97–1.03)	1.01 (0.98–1.04)	0.97 (0.95–1.00)
*p*-Value				<0.001	0.939	0.420	0.041
AMD without VD	36,003	4684	15.7	1.16 (1.12–1.19)	1.00 (0.97–1.03)	1.01 (0.98–1.04)	0.97 (0.94–1.00)
AMD with VD	2723	375	16.9	1.25 (1.13–1.38)	1.06 (0.95–1.17)	1.06 (0.96–1.17)	1.03 (0.93–1.14)
^†^*p* for trend				<0.001	0.756	0.312	0.087

IR, incidence rate; PY, person-years; HR, hazard ratio; aHR, adjusted hazard ratio; CI, confidence interval. ^†^ *p* for trend was calculated by the (adjusted) hazard ratios among the control, AMD without VD, and AMD with VD groups. Model 2 was adjusted for age and sex. Model 3 was adjusted for age, sex, income, area of residence, body mass index, smoking, alcohol consumption, and regular exercise. Model 4 was adjusted for age, sex, income, area of residence, body mass index, smoking, alcohol consumption, regular exercise, fasting serum glucose level, serum hemoglobin level, eGFR, hypertension, dyslipidemia, and CCI.

## Data Availability

The datasets used and/or analyzed during this study are available from the corresponding author on reasonable request.

## Supplementary Materials

The following supporting information can be downloaded at: https://www.mdpi.com/article/10.3390/biomedicines10102435/s1, Table S1: Visual disability criteria of the Republic of Korea.
