# Supplementary material for: Association between Age-Related Macular Degeneration and the Risk of Diabetes Mellitus: A Nationwide Cohort Study
Source: Biomedicines. 2022 Sep 29;10(10):2435. doi: 10.3390/biomedicines10102435 (PMC9599121; doi:10.3390/biomedicines10102435)
Supplement: Supplementary file 1 [file biomedicines-10-02435-s001.zip › biomedicines-1912325-supplementary.pdf]

Supplementary Table S1 Visual disability criteria of the Republic of Korea.

Grade of Visual disability	Criteria
1 (most severe)	BCVA ≤ 0.02 in the better eye
2	BCVA ≤ 0.04 in the better eye
3	1) BCVA ≤ 0.06 in the better eye, or 2) bilateral visual field < 5 degrees in all perspectives
4	1) BCVA ≤ 0.1 in the better eye, or 2) bilateral visual field < 10 degrees in all perspectives
5	1) BCVA ≤ 0.2 in the better eye, or 2) Decrease in bilateral visual field of 50% or more from the normal
6 (least severe)	BCVA ≤ 0.2 in the worse eye

BCVA, Best-corrected visual acuity.
